# Mesocosm experiments reveal the impact of mosquito control measures on malaria vector life history and population dynamics

**DOI:** 10.1038/s41598-018-31805-8

**Published:** 2018-09-17

**Authors:** Kija Ng’habi, Mafalda Viana, Jason Matthiopoulos, Issa Lyimo, Gerry Killeen, Heather M. Ferguson

**Affiliations:** 1 Ifakara Health Institute, Environmental Health and Ecological Sciences, Ifakara, United Republic of Tanzania; 2 Institute of Biodiversity, Animal Health and Comparative Medicine, College of Medical, Veterinary and Life Sciences, University of Glasgow, Glasgow, G12 8QQ United Kingdom; 3 Liverpool School of Tropical Medicine, Department of Vector Biology, Liverpool, United Kingdom; 4 School of Health Sciences, University of Dar es Salaam, Dar es Salaam, Tanzania

## Abstract

The impact of control measures on mosquito vector fitness and demography is usually estimated from bioassays or indirect variables in the field. Whilst indicative, neither approach is sufficient to quantify the potentially complex response of mosquito populations to combined interventions. Here, large replicated mesocosms were used to measure the population-level response of the malaria vector *Anopheles arabiensis* to long-lasting insecticidal nets (LLINs) when used in isolation, or combined with insecticidal eave louvers (EL), or treatment of cattle with the endectocide Ivermectin (IM). State-space models (SSM) were fit to these experimental data, revealing that LLIN introduction reduced adult mosquito survival by 91% but allowed population persistence. ELs provided no additional benefit, but IM reduced mosquito fecundity by 59% and nearly eliminated all populations when combined with LLINs. This highlights the value of IM for integrated vector control, and mesocosm population experiments combined with SSM for identifying optimal combinations for vector population elimination.

## Introduction

Vector control remains the best way to reduce transmission of malaria and other mosquito-borne infections^1^. Quantification of the specific and combined impacts of control measures on vector demography is particularly valuable for the goal of population elimination. Such measurements can highlight thresholds for tipping populations into extinction trajectories, estimating their rebound capacity after suppression, and validating whether interventions are acting as anticipated^2^. Population dynamics models are routinely applied to guide management decisions in other areas of ecological management (e.g.^3,4^). Whilst mathematical modelling is widely used for guiding malaria control policy^5^; there has been relatively limited use of population dynamic models to guide mosquito control strategies (though see^6,7^). The potential benefits of population dynamics-based approaches to vector control are particularly relevant for human malaria, which causes greater human mortality and morbidity than any other vector-borne disease. While the scale-up of vector control measures has proven extremely successful, current front-line methods have limitations meaning that they are unlikely to achieve malaria elimination on their own^8–10^.

Long-lasting insecticidal nets (LLINs) are highly effective, widely used and affordable vector control tools for malaria prevention in Africa. The scale-up of LLINs across Africa has made a substantial contribution to the ~68% of reduction in malaria cases^8^ and 57% reduction in malaria mortality achieved since 2000^9^. LLINs can directly prevent human exposure through physical protection, and also generate a community protection effect by killing mosquitoes and reducing their population size and transmission potential^1^. However, LLIN effectiveness relies on the behavioural predisposition of malaria vectors to feed predominantly on humans indoors at night when they are in bed. The near elimination of the vector *Anopheles gambiae* from several African settings after LLIN introduction has been attributed to this species’ near exclusive indoor, human biting behaviour^2,11^. However, residual malaria transmission in these settings is often maintained by more behaviourally-plastic vectors such as *An*. *arabiensis* that can feed outdoors (exophily), on non-human host such as cattle (zoophily)^12^, and enter and exit houses without making fatal contact with treated surfaces^13^.

Widening the range of control approaches to include those that can target mosquito vectors with diverse behaviours and at multiple points in their life cycle is required for tackling residual transmission^10,14,15^. However, it remains unclear which suite of interventions will be most effective for suppressing, and ideally eliminating malaria transmission^2^.This partly stems from our limited understanding of the different ways in which existing interventions can influence mosquito demographic rates. For example, while LLINs target the survival of adult female mosquitoes, they may also have indirect impacts on their reproductive rate by reducing their probability of blood feeding, and increasing the time required to produce eggs^1^. Also, impacts of LLINs on one life-history trait may trigger compensatory increases in another due to underlying density dependence. Control measures that kill vectors and reduce their population size may indirectly increase the per capita fecundity of survivors because of reduced competition in larval habitats^7,16,17^. Additional complexity arises when interventions are combined that could potentially enhance or counteract their respective, independent impacts. For example, there is interest in intensifying the coverage of insecticides inside houses by combining the use of LLINs with Indoor Residual Spraying^18,19^ and/or the application of insecticides to other house entry points (e.g. eaves^20–22^). It is hypothesized that such combinations would be more effective than LLINs alone because they could further target mosquitoes that enter houses, but would otherwise escape without making contact with them^23^. However, such combinations may yield little additional impact if their impacts overlap, or if they are used in settings where most residual transmission is due to outdoor biting. Clearly, there is a need to understand both the independent and coupled impacts of interventions to evaluate which combinations hold most promise.

Measuring the impact of specific interventions on vector fitness (e.g. survival and reproduction) and life-history is challenging in the field because direct measurement of these traits in wild populations is not feasible. Ovariole dissections^24^ are commonly used to estimate survival on the basis of whether a mosquito has laid eggs or not, but these estimates are coarse, biased and require making assumptions about life history processes. Alternatively, studies of mosquito fitness in response to interventions can be conducted in small-cage laboratory settings but their relevance and scalability to natural populations is unclear. Experimental mesocosms have long been used in ecology to strike a balance between the artificial conditions of laboratory studies, and the expense and difficulty of conducting more realistic assessments in the field. A mesocosm is defined as a closed experimental system in which the key features of the natural environment are present. Mesocosm populations are contained, so they can also provide estimates of life-history and demographic rates that are unaffected by dispersal^25^. These systems enable replicated, population-level experiments to be conducted, with a close degree of population monitoring that would not be achievable or affordable in field studies. Mesocosms have been used for experimental study of population dynamics in a wide range of species and communities^26–30^. Recently, progress has been made with the development of simple semi-field systems for study of mosquito vector ecology and behaviour^31–34^; including demonstration that self-propagating mosquito populations can be maintained over multiple generations^32^ while retaining natural behaviour, life history and levels of genetic diversity^34,35^. However, so far these systems have been mostly used for short-term experiments^36^, rather than longer-term assessment of how vector population dynamics may respond to interventions.

Several new control measures are under consideration for vectors of residual malaria transmission^37^. Among these options, the veterinary systemic endectocide Ivermectin (IM) is receiving considerable attention due to its potential for targeting mosquitoes that feed outdoors and on animals as well as humans^38–41^. IM is frequently given to cattle for treatment of intestinal helminths, and to people as a treatment for onchocerciasis and filariasis^42,43^. Clinical doses of IM in host blood have been shown to reduce the survival and fecundity of mosquitoes that feed on it in laboratory and semi-field bioassays^44–46^. Similarly, mass drug administration of ivermectin to people has been associated with reduced mosquito survival, parity rates^47^ and malaria prevalence^47,48^ in the field. This ability to impair multiple mosquito fitness traits and behaviours could make IM a promising strategy for controlling exophilic vectors responsible for residual transmission. However, it remains unclear whether the effects of IM administrated to cattle would scale to mosquito population-level impacts, and how they would interact with other control measures such as LLINs. Robust evidence on the consequences of such measures on mosquito fitness and population dynamics is needed.

**Figure 1:**
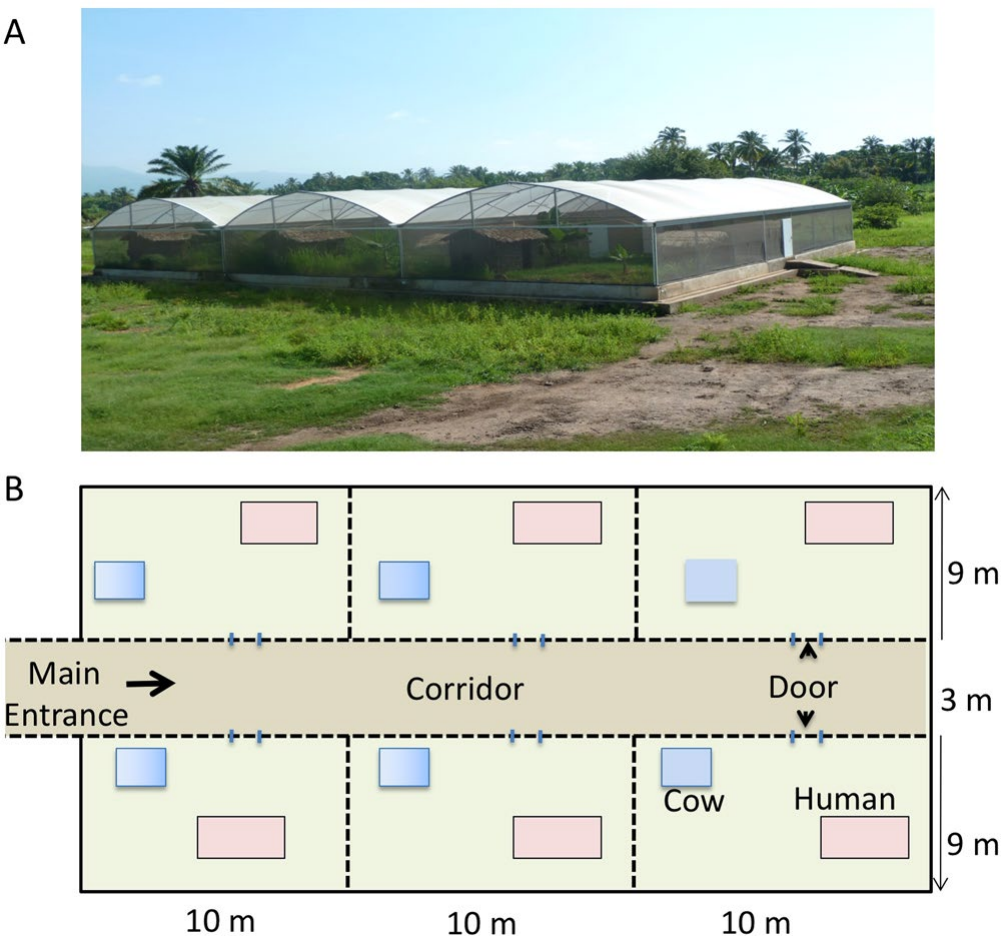
(**A**) Side view one of the two mosquito semi-field systems (SFS) situated near Ifakara, Tanzania in which mesocosm experiments were conducted. Each SFS contained 6 replicate mescosm chambers (12 in total) with *An*. *arabiensis* populations being successfully established in 9 chambers. (**B**) Schematic layout of one SFS, showing the position and size of each mesocosm chamber.

**Figure 2:**
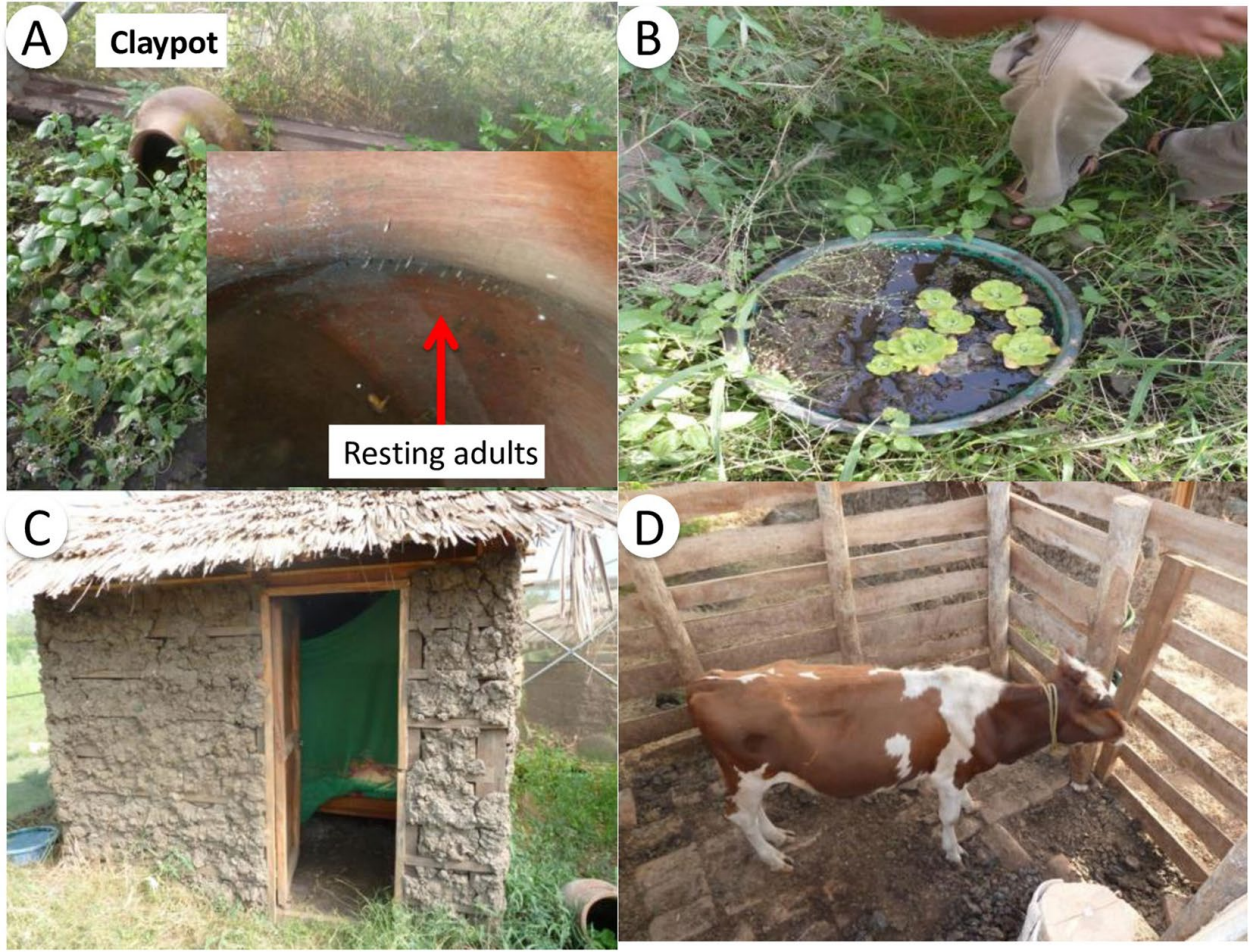
Habitat features within each mesocosm chamber (**A**) The inside of a clay pot which adult mosquitoes used for resting, (**B**) Larval habitat created using plastic buckets (**C**) house built in local style under which human volunteers slept (here shown with LLIN), (**D**) shed in which cattle hosts (calves) were held overnight.

Here, we describe the use of a unique large-scale mesocosm facility in southern Tanzania^34,35^ to investigate the response of malaria vector populations to perturbation by combinations of vector control methods (Figs 1 and 2). Replicated populations of *An*. *arabiensis*, a vector of residual transmission across Africa^13^, were exposed to LLINs either on their own or combined with two other types of intervention strategies: (i) treatment of cattle with IM or (ii) the further application of insecticides to houses through installation of insecticide-treated eave louvers (ELs). Data on the density of adult mosquitoes and larvae collected in these mesocosms were used to fit a state-space population model which quantified the impacts of different combinations of interventions on *An*. *arabiensis* population stability. This approach highlights hidden vulnerabilities in vector life history processes that can be targeted by using complementary combinations of vector control tools to precipitate dramatic population collapse.

## Results

**Figure 3:**
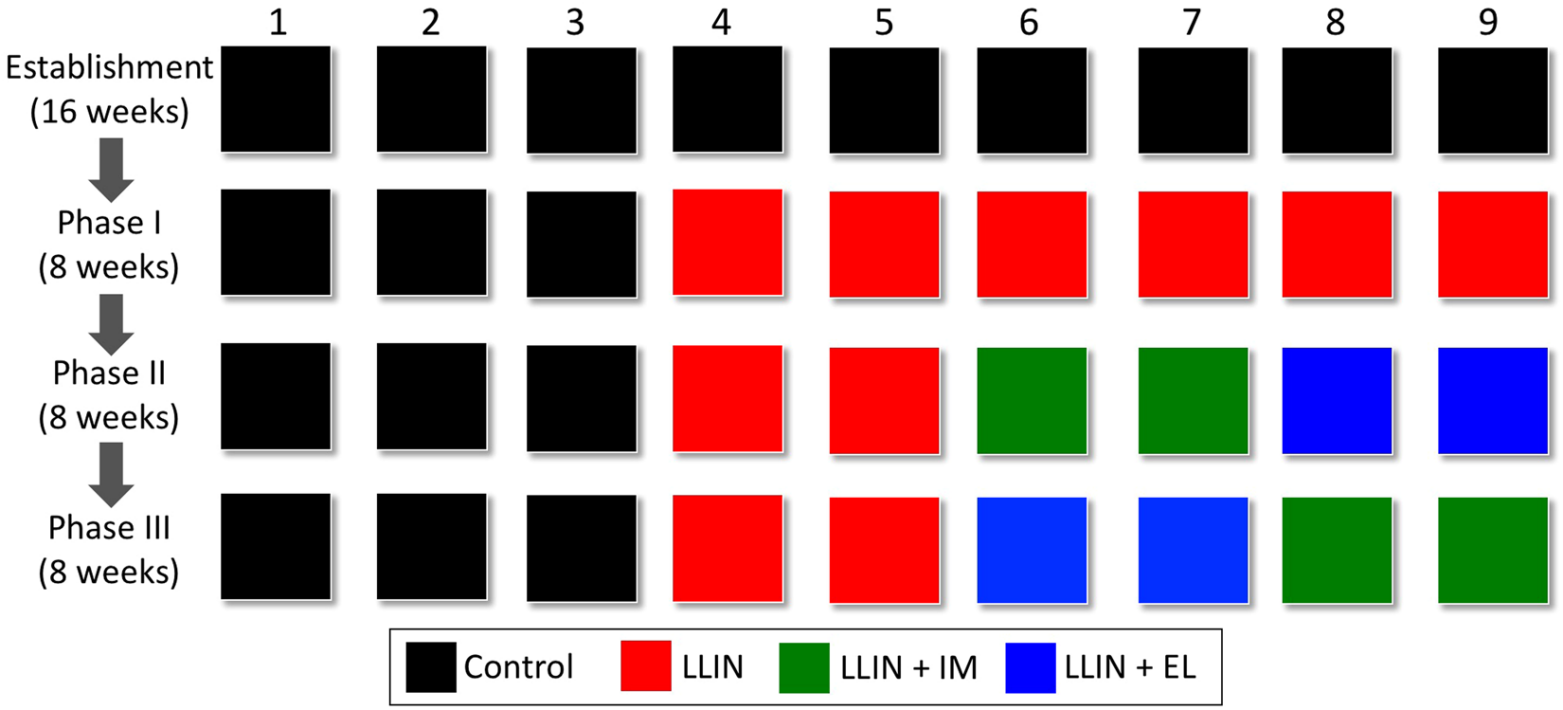
Diagram of the experimental design showing the types of intervention (different colours) implemented in each of the 9 mesocosm chambers (columns) across the different phases of the experiment (rows).

Nine replicate populations of the malaria vector *An*. *arabiensis* populations were established in large mesocosm chambers and allowed to stabilise in the absence of interventions as described in Fig. 3. To allow blood-feeding and reproduction, mosquitoes had access to one human and one cow host for 5 nights each week (Figs 1 and 2). After establishment, LLINs were introduced into 6 of the 9 populations, with 3 remaining intervention-free to act as controls. This *Phase I* lasted for 8 weeks, after which IM was administered to cattle within 2 of the LLIN-chambers, and insecticide treated ELs were installed in the houses of other 2 LLIN-chambers (*Phase II*). In the subsequent *Phase III*, IM and EL treatments were swapped between chambers for a final 8 weeks (Fig. 3). Mosquito surveillance was conducted by sampling larvae and adult females every 2–4 weeks in these chambers until the end of the study (using a larval dipper in aquatic habitats, and Human Landing Catches; further details in Methods and Supplementary Information).

**Figure 4:**
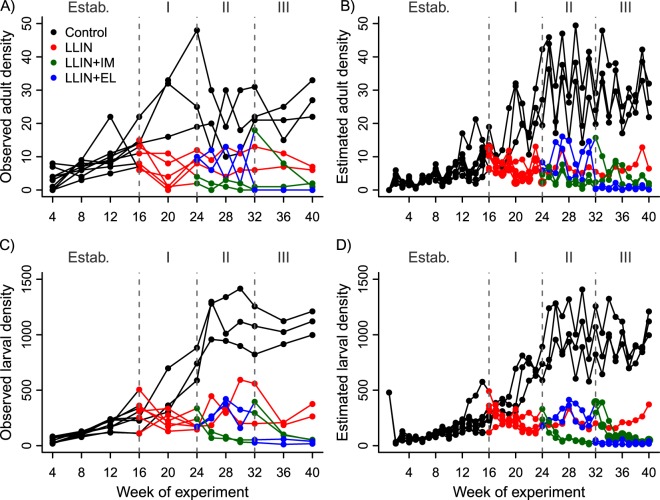
Observed (left column) and estimated (right column) mean abundance of adult female (top row) and larval *An*. *arabiensis* (bottom row) mesocom populations during the experiment. Black lines correspond to control populations (not exposed to any intervention), red lines to populations exposed to long lasting insecticidal nets (LLINs), green lines to populations exposed to LLINs and Ivermectin (IM), and blue lines to populations exposed to LLINs and eaves louvers (EL). See Table S2 for summary of estimated mean densities and associated 95% credible intervals.

**Figure 5:**
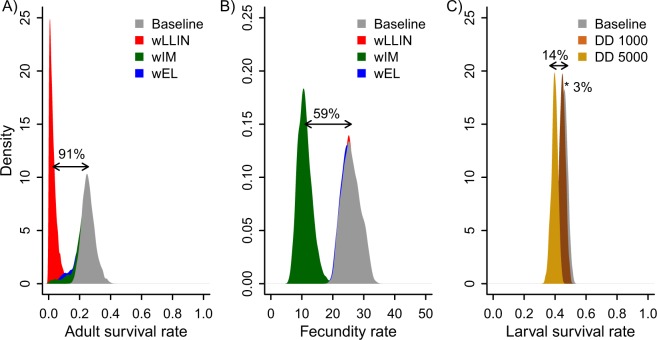
Posterior density distributions of the key demographic parameters under the pressure of different interventions obtained from the state-space population model: weekly (**A**) adult survival rate baseline (control; black) and impacted by LLINs (red), EL (blue) or IM (green); (**B**) fecundity rate (number of 1^st^ instar larvae/week) baseline (control; black) and were impacted by LLINs (red), EL (blue) or IM (green); and (**C**) larval survival rate baseline (control; black) and impacted by density-dependence (DD) when the population size is 1000 or 5000 individuals. The percentages correspond to the estimated weekly reductions in baseline (survival and fecundity) rates driven by the corresponding intervention or DD. Note: some densities are overlaid on the baseline due to little impact of the corresponding intervention.

Following the initial introduction of mosquitoes into mesocosms, *An*. *arabiensis* populations began to grow. In the control chambers, both larvae and adult females increased until approximately week 26 when numbers stabilized (Fig. 4). The population trajectory within the control chambers (ie. all chambers prior to intervention introduction, and those that never received an intervention) was estimated to arise from a baseline median adult female survival of 0.25 (with credible interval of 95% central quantile, hereafter 95% CI = 0.18, 0.35) per week (Fig. 5A), and a baseline fecundity of 26 larvae per female each week (95% CI: 20.9, 32.5 larvae; Fig. 5B). The median baseline of larval survival was estimated as 0.46 per week (95% CI: 0.42, 0.50; Fig. 5C). These weekly rates correspond to a daily survival of 0.89 for larvae and 0.82 for adults Additionally there was clear evidence of density-dependence at the larval stage, with larval survival being reduced by 2.8% per week (95% CI: 1.5, 4.6%) per 1000 individuals or 13.6% (95% CI: 7.2, 22.3%) per 5000 individuals (Fig. 5C, brown and yellow densities, respectively) in the control population.

All three intervention treatments (LLINs, LLINs + EL, LLINs + IM) led to reductions in larval and adult mosquito densities compared to the controls (Fig. 4). The SSM was used to quantify the changes in mosquito life history traits (survival and fecundity) that would be required to give rise to these treatment-specific population dynamics. The model converged well (Gelman-Rubin point estimate below 1.0^49^;) and reconstructed the population dynamics of both larvae and adult mosquitoes satisfactorily (Fig. 4: comparison between left and right panels; Figure S3: shows observed versus predicted values).

The introduction of LLINs at the start of *Phase I* altered this population growth trajectory in all chambers where they were allocated. In contrast to the control chambers where mosquito numbers continued to rise, population growth immediately halted in the presence of LLINs (Fig. 4, left panels). On the basis of these dynamics, the introduction of LLINs was estimated to reduce the weekly adult female survival rate by ~91% (95% CI: 60–100%; Fig. 5A and Table 1) relative to the controls. The observed population dynamics indicated that LLINs had negligible impact on mosquito fecundity (estimated median reduction of 0.003% larvae per week, 95% CI: 0, 3.8%; Fig. 5B). Although the introduction of LLINs significantly suppressed *An*. *arabiensis*, their populations were able to persist in their presence until the end of the experiment (Fig. 4).

**Table 1:** Estimated median (95% CI) life history rates (adult survival and fecundity) and degree of population suppression achieved in experimental mesocosms with different types of interventions (LLINs: Insecticide-treated bednets; IM: Ivermectin; EL: Eaves louvers).

Intervention	Estimated adult female survival (weekly)	Estimated adult female fecundity (1^st^ instar produced per week)	% Pop. suppression (relative to controls)	% Pop. suppression (relative to LLINs only)
Control (baseline)	0.25(0.18–0.35)	25.77(20.94–32.42)	N/A	N/A
LLINs	0.02(0.00–0.10)	25.65(20.81–32.37)	73.85(68.36–78.73)	N/A
EL	0.24(0.08–0.34)	25.63(20.73–32.26)	93.67(89.77–96.56)	19.82(21.40–17.83)
IM	0.24(0.07–0.34)	10.76(7.12–16.54)	99.48(98.11–100)	25.62(29.74–21.27)

Note that population suppression values for EL and IM represented the combined effects arising when used with LLINs (LLINs+IM or LLIN+EL) and that EL and IM treatments were swapped in all chambers after 8 weeks, so values correspond to suppression in populations where the specific intervention (left hand column) was introduced first. Population suppression values are given relative to control populations in mesocosms where no interventions were present, and in others where only LLINs were in place.

The introduction of insecticide-treated ELs to houses in chambers where LLINs were already in use had little additional impact on *An*. *arabiensis* populations (Fig. 4; Table 1). The model estimated adult survival and fecundity rates to be similar in the LLIN-only and LLIN-EL chambers (Fig. 5A,B, blue densities overlapping with control grey densities). In contrast, supplementing LLINs with IM had a significant additional impact on *An*. *arabiensis* populations. Notably, the two populations where IM was introduced in addition to LLINs crashed within 4 weeks (Fig. 4). While IM was estimated to have negligible incremental impact on adult mosquito survival (reduction of 0.1% compared to controls; 95% CI: 0, 7%; Fig. 5A), it triggered an additional 59% reduction (95% CI: 41, 70%) in the median weekly fecundity of *An*. *arabiensis* (compared to the controls and LLIN-only treatments, Fig. 5B).

Overall, the combination of LLINs followed by IM had the most disruptive effect on vector populations. Introduction of LLINs reduced the adult population by 74%, resulting in a predicted density of only 10 adult females and several hundred larvae in LLIN-populations (Table S2). The population densities in chambers where ELs were combined with LLINs were no different than those with LLIN alone (Table S2). However, adult female mosquitoes dropped almost below detection (n = 0–3; Table 1) within 8 weeks in chambers where LLINs were combined with IM. Despite the considerably lower adult densities in LLIN + IM chambers, a small number of larvae were predicted to persist (≤50,Table S2). Whilst the continued detection of a few larvae in LLIN + IM treatments indicates that population elimination was not achieved, these populations would be at high risk of stochastic extinction. However, even if neither larvae nor adults were observed we could not assume elimination because our model indicated that their respective detection probabilities (i.e. estimated proportion of the population observed through sampling) were 15% and 10%.

## Discussion

We combined experimental manipulation of replicated mosquito populations within mesocosms and Bayesian state-space models to estimate the impacts of the current frontline malaria vector control strategy (LLIN) plus supplementary approaches (EL and IM) on the fitness and population dynamics of the major African vector *An*. *arabiensis*. This approach allowed us to quantify the degree to which distinct mosquito life history processes (adult survival and fecundity) were affected by interventions, and estimate their combined impact on vector population stability. Typically the impacts of interventions upon mosquito vector fitness are estimated from direct observation in simplified laboratory bioassays and/or indirect measurement in the field^50^. Simulation models often use this type of data to predict the likely impact of these interventions on mosquito populations and malaria transmission^7,51,52^. While such approaches are clearly valuable for focussing research efforts, their accuracy may be limited by the reliability of mosquito fitness and behaviour data on which they are based. Estimates of mosquito fitness obtained from individuals or cohorts in bioassays may not reliably scale to population-level responses. Similarly, the few direct estimates of mosquito fitness that can be measured in the field (e.g. parity rates) are not very precise and subject to bias. Here, we present the use of mesocosm population experiments paired with SSMs as a strategy for refining and improving estimation of the population-level response of mosquito vectors to interventions. Large-scale randomized field studies will always remain the gold standard for evaluating the impacts of interventions^53^, but the considerable time and investment required for such studies are only warranted for the most promising strategies. An advantage of the approach developed here is that it allows the impacts of interventions on mosquito fitness to be predicted at a population scale under realistic environmental conditions.

Our study confirms the expectation that the main direct mechanism of action of LLINs is a reduction in adult mosquito survival^54^, with little subsequent impact on the fecundity of survivors. Despite the known ability of *An*. *arabiensis* to avoid lethal contact with LLINs^23^, coverage of human hosts with LLINs within these systems was sufficient to substantially reduce, but not eliminate, *An*. *arabiensis* populations.The high weekly mosquito mortality predicted here is likely due to LLIN coverage being unrealistically fixed at 100% in these experiments, with all available human hosts being underneath nets from ~7 pm–7am. In contrast, community members in surrounding areas typically do not go inside to sleep until 9 pm or later^55^, thus they are exposed to biting in the early evening. Whilst it is not feasible to change the time that people go to bed, this study demonstrates the substantial reductions in malaria vector populations that could be achieved by combining LLINs with another strategy that protects people from bites outdoors^37^. There are several potential explanations for why LLIN introduction did not have any impact on mosquito fecundity. For example, perhaps the mosquitoes that were able to feed on humans in the presence of LLINs (e.g. by entering through holes) had no feeding impairment, or mosquitoes switched to feeding on cows when LLINs were introduced. These hypotheses cannot be distinguished in this experimental design. However, the overall finding of *An*. *arabiensis* persistence despite the large increase in adult mortality highlights the considerable challenge of achieving vector population elimination using only LLINs.

This study provides grounds for optimism that even behaviourally evasive species like *An*. *arabiensis* could be effectively suppressed or even eliminated using appropriate combinations of vector control interventions^2^. Here, we focussed on two different approaches for combining interventions: the first based on intensifying the coverage of insecticides in houses by combining LLINs with ELs, and the second on targeting mosquitoes that feed outside of houses and on other animals by treating cattle with IM. In this comparison, extending intervention coverage to an alternative host (e.g. IM on cattle) rather than applying additional protective methods to housing (EL) appeared most effective. This outcome was not guaranteed, as other studies indicate that *An*. *arabiensis* can enter and exit houses without making fatal contact with LLINs^13^, and significant benefits can be obtained by targeting house entry points and walls with insecticides^18–22^. Whilst the particular EL design used here had little impact on *An*. *arabiensis*, other studies using different designs of insecticide-treated materials, such as curtains, eave baffles and eave tubes have shown more promise^20–22^. Consequently the value of approaches that seek to further target mosquito vectors entering houses should not be dismissed, and may have considerable impact depending on the details of physical design, materials and insecticide, as well as the behaviour of target vector species.

Treatment of cattle with IM in mesocosms where the only other hosts were humans (protected by LLINs) had no additional impact on *An*. *arabiensis* adult survival, but triggered a significant decline in their fecundity. In previous studies using the same dosage as this study, IM in cattle was found to reduce *Anopheles* survival and egg production^56,57^. Our estimates of the population-level consequences of IM differ from these results based on individual-level bioassays in two important ways. First, we failed to detect any additional impact of IM on mosquito survival at the population level, in the presence of LLINs. As there were relatively few adult mosquitoes remaining in mesocosms when IM was introduced and the sampling frequency was relatively coarse (every 2 weeks), it is possible there was low statistical power to detect any additional small-to-moderate impacts that IM could have had on mosquito survival. However studies of *An*. *arabiensis* held in small cages indicates ivermectin causes large reductions in survival (>50% mortality between gonotrophic cycles^41^. Second our estimate of the impact of IM on mosquito fecundity (e.g. mean number of larvae produced per week) is lower than what would be extrapolated from previous semi-field bioassays^56^. In these bioassays, *An*. *arabiensis* were allowed to feed on an IM-treated cattle in an mesocosm then captured and individually held in a tube to record oviposition rates and egg production. Here, IM was associated with a ~67% reduction in the proportion of mosquitoes laying eggs, and a further 62% reduction in the number of eggs produced by those that laid^56^. In combination these processes would be expected to reduce mean offspring production by ~88% in contrast to the 59% predicted here. While it would have been useful to measure the egg production of mosquitoes in these experiments for comparison with these previous studies, we caution that this in itself would not provide a meaningful validation because individual-level fecundity cannot be assumed to scale reliably to population level recruitment as was our aim. Further studies will be required to identify why the impact of IM measured at the mosquito population level here differs from that estimated in individual bioassays. However, when combined with LLINs, the reductions in mosquito fecundity achieved from IM were sufficient to crash all experimental *An*. *arabiensis* populations to barely detectable levels within a few weeks of introduction. This adds to the growing support for the use of veterinary formulations, and IM in particular, as a means to tackle residual malaria transmission^39,40^.

In addition to elucidating the impacts of interventions, this study provides insights into the fundamental basis of malaria vector population dynamics. Laboratory studies indicate that larval density in aquatic habitats is inversely related to their development rate and adult fitness^58,59^. These effects govern the density-dependent population growth reported in wild populations^16,17^. We acknowledge that the ephemeral nature of wild larval habitats and potential larval predators are not captured in these simplified mesocosms, and that this could result in the overestimation of density-dependent larval mortality here. Bearing this in mind, we observed that the inclusion of density-dependent larval mortality substantially improved the fit of our SSM to the observed dynamics of *An*. *arabiensis* in these systems. This overall finding strengthens evidence that larval competition plays an important role in vector population regulation and the outcome of control measures^60,61^.

The growing recognition of the importance of vector population dynamics to intervention outcomes^62,63^ has raised interest in refining models to incorporate vector demography (e.g^7,64–66^). The Bayesian SSM framework applied here illustrates the potential of this approach when combined with replicated mesocosm systems. Our approach also enables extensions for the description of the demographic rates. For example, in open systems, covariates for temperature and rainfall could be fit as drivers of reproduction and larval development within this framework, as could interactions between interventions if their simultaneous use is expected to change their independent effects. An additional advantage of the SSM framework is that it permits explicit characterisation of biases and imprecision inherent in the data collection process. For example, it was possible to estimate the proportion of the mosquito population observed on each sampling occasion (<15% for larvae and adult females). The inability to account for these sampling biases is a longstanding challenge in animal ecology, where it limits accurate measurement of population size in open field studies^67^. Explicit incorporation of error and bias in the observation process could significantly improve the value extracted from standard entomological surveillance, and its use for inferring vector population dynamics.

This study has some notable limitations. While the mesocosms used here incorporated significantly more environmental realism than laboratory studies, they differ from the field in several important ways. The mosquito populations were enclosed and not subject to dispersal, genetic exchange, predator-prey interactions and migration processes which could dampen the population peaks and crashes observed here. Additionally, although exposed to natural temperature and climate conditions, mosquitoes inside the mesocosms were protected from rainfall. Seasonal variation in rainfall generates substantial fluctuations in malaria vector population size, which could either attenuate or increase impacts observed here^68^. Furthermore, only one human and cow host was presented to mosquitoes in each chamber, making it possible to achieve 100% “biological coverage” (e.g. as defined in^69^) of all potential hosts with an intervention. Achieving this level of coverage is likely impossible under realistic programmatic conditions. Furthermore we highlight that in these experiments, mosquito populations may not have fully stabilized when LLINs were introduced. This could have resulted in overestimation of their impact on mosquito survival in earlier phase if the control populations were still rising. However, as the impacts here are estimated relative to the mean survival of the control over the entire experiment, we expect these effects would be averaged out. Finally, we only measured mosquito vector dynamics, and not it’s associated impact on malaria transmission or disease burden. Although mosquito vector traits such as adult survival and abundance are important drivers of transmission, the evidence base for policy recommendation of new vector control tools will ultimately require rigorous epidemiological evidence^53^.

## Conclusions

Here, we describe a robust approach that combines mesocosm population experiments and process-explicit ecological models to highlight the distinct ways that vector control interventions can perturb mosquito vector populations. Specifically, our findings highlight the added value of veterinary ivermectin formulations, one of several promising new interventions becoming available to tackle residual malaria transmission^37^. This ability to experimentally quantify the impact of interventions on mosquito fitness at the population level constitutes a major advance in our ability to identify which combinations may be most effectively used for mosquito elimination. Furthermore, this study provides proof-of-principle of the substantial additional insight that could be extracted from mosquito surveillance data using integrated population models. Similar techniques could be extended to natural populations to provide a framework for predicting the complex impacts of interventions, and ultimately deciding which combinations will be most effective for going beyond control of vector populations to elimination^2^.

## Methods

### Mesocosm experiments

Two large (726 m^2^) semi-field systems were built in Ifakara, southern Tanzania^35^, each compartmentalized into 6 identical 90 m^2^ self-contained mesocosm chambers (Fig. 1A). Independent populations of *An*. *arabiensis* were established in these mesocosm chambers as described previously^32,34,70^. Features of the local environment were recreated in each mesocosm chamber (Figs 1 and 2). Each chamber contained a mud walled house, local vegetation, 10 larval habitats made using plastic buckets (diameter 43.5, depth 5 cm) which were buried to ground level and filled with water, and 10 locally-made clay pots which acted as mosquito resting sites.

### Establishment of *An. arabiensis* populations inside mesocosm chambers

From October 2012 to February 2013, blood-fed female mosquitoes identified morphologically as members of the *An*. *gambiae* complex were collected from Sagamaganga village, and transported to the Ifakara Health Institute. A baseline survey to detect presence/absence of pyrethroid resistance within this founder population was conducted in 2012. Here, more than 80% of *An*. *arabiensis* died in the 24 hours following exposure to alphacypermthrin and deltamethrin, indicating the population was susceptible (*Issa Lyimo*, *pers comm*.). First generation eggs from field-caught females confirmed as being *An*. *arabiensis* by polymerase chain reaction^71^ were pooled and released into aquatic habitats to establish populations under semi-field conditions (see Supplementary Information for more details). Human volunteers and calves (1 of each) were made available to mosquitoes within each chamber for 5 nights each week. Hosts stayed in each chamber from 7.00 pm to 7.00 am each day. When spending the night inside a mesocosm, volunteers slept on a bed inside the house (Fig. 2C), and cattle were tethered inside a corral with access to food and water (Fig. 2D). Human volunteers rotated between mesocosms each night. Cattle were randomly assigned to mesocosms at the start of the experiment and stayed in the same one throughout.

The *Establishment phase* lasted for 16 weeks, during which the number of larvae and pupae in each aquatic habitat was surveyed once per month by counting the number sampled within a single ~350 ml scoop of water with a standard larvae dipper from a total volume of ~3 L per habitat. Collected larvae/pupae were released back into larval habitats. Adult female abundance was assessed by conducting human landing catches (HLC) in mesocosms once per month. Two people performed these collections, one sitting inside the house, and the other sitting just outside it. Both individuals exposed their lower legs and collected mosquitoes attempting to bite them from 7.00 pm to 7.00 am. Mosquitoes captured in HLCs were counted and taken to the laboratory for storage. At the end of this establishment phase, populations were successfully established in 9 out of the 12 mesocosm chambers.

### Experimental Design

A diagram of the experimental design is shown in Fig. 3. In the first step (*Establishment phase*), replicate *An*. *arabiensis* populations were established in 9 mesocosm chambers and allowed to equilibrate in the absence of interventions. After establishment, populations were exposed to a series of interventions that were introduced sequentially to evaluate: (1) how vector fecundity, survival and population abundance were impacted when human hosts started using LLINs, and (2) the additional impact of introducing one of two prospective complementary interventions, insecticide-treated ELs, or systemic IM treatment of cattle. The impacts of EL and IM were evaluated only in combination with LLINs, and not on their own. This decision reflects the most relevant baseline for evaluating new control measures in Africa, where ~80% of households have access to at least one LLIN^72^ and it is unlikely that any new intervention would be deployed in their absence.

In the first phase of the study (*Phase I*), human volunteers sleeping in 6 of the 9 mesocosm chambers were protected when they slept by hanging a LLIN (Permanet 2.0^®^, Deltamethrin 42–55 mg/m^2^) over their beds. The remaining 3 chambers were kept intervention-free as controls. This *Phase I* lasted for 8 weeks, after which two additional interventions were introduced into 4 out of the 6 LLIN-chambers (*Phase II*) for another 8 weeks. In *Phase II*, IM (1% ivermectin solution) was administered to cattle within 2 of the 6 LLIN-chambers by subcutaneous injection (1 mg/5 kg of body weight = 0.2 mg/Kg once every 4 weeks). In another 2 LLIN-chambers, insecticide treated ELs were installed in the houses along the eave gap between the top of the walls and the roof (details in SI). As a strategy for limiting the impact of pseudoreplication arising from the potential confounding of “treatment” with experimental chamber, IM and EL treatments were swapped between chambers for a final 8 weeks (Phase III).

### Vector population monitoring

Throughout this 42-week experiment (*Establishment phase* to end of *Phase III*), the abundance of larval and adult female mosquitoes in each mesocosm population was assessed every 4 weeks, except for *Phase II* when it was conducted every 2 weeks. Larval and adult females were counted as described for the Establishment Phase.

### Ethical considerations

All methods for housing livestock and administering ivermectin to cattle were carried out under the supervision of a veterinarian in accordance with relevant guidelines and regulations. All methods that involved human volunteers were performed in accordance with the relevant guidelines and regulations. Ethical approval for these procedures was obtained from the Ifakara Health Institute Institutional Review Board (IHI/IRB/No. 21–2010) and the Tanzanian National Institute for Medical Research (NIMRlHQ/R.8a/Vol. IX/708). Written informed consent was provided from members of the research team who volunteered to sleep in the mosquito mesocosm chambers and conduct human landing catches.

### State-space population model

To quantify the impact of control strategies on the different life stages of *An*. *arabiensis*, a population model was developed within a Bayesian state-space modelling (SSM) framework. The foundation of this model was the mosquito life cycle (i.e. adult female mosquitoes blood feed then lay eggs, which hatch into larvae, pupae and then adults), with females producing a new generation of eggs. Larval development from egg to pupae typically takes 7–10 days, with the pupal stage lasting ~48 hours^32,73^. We did not explicitly model larval and pupal stages separately, but rather aggregated them into two one-week long development stages so as to coincide with the timescale of data collection. These stages can be broadly defined as early (week 1: egg to 2^nd^instar larvae) and late-stage (week 2: 3^rd^, 4^th^ instars and pupae) development.

Full details of the model are provided in SI, with a brief summary here. Specifically, adult female survival was modelled as a binomial process with a weekly survival probability expressed as a function of covariates. Adult survival probability *S*_*p*_(i,t) in each chamber *i* at each one-week time step *t*, was formulated as a logit function of the three intervention treatments:

1 $$logit({S}_{p}(i,t))={\beta }_{0}-{\beta }_{1}IT{N}_{i,t}-{\beta }_{2}I{M}_{i,t}-{\beta }_{3}E{L}_{i,t}+{\varepsilon }_{i,t}$$

where β_0_ determines the baseline mean weekly adult female survival, and the parameters β_1_, β_2_ and β_3_ quantify the impact of LLINs, IM or EL respectively. Note that the impact of each intervention is estimated relative to the baseline survival, i.e. mean survival of the control group. The error term $\varepsilon$ accounts for variability in the biological process that is not accounted for by simple binomial (demographic) stochasticity.

The number of adult females (*p*_*i*,*t*_) that survive and lay eggs which develop into 1st instar larvae, with a weekly per-capita fecundity rate *b*_*i*,*t*_ and the number of eggs that develop into female larvae (on assumption of 50:50 sex ratio) was modelled as Poisson process (${n}_{{0}_{i,t+1}} \sim Poisson({b}_{i,t}{p}_{i,t})$. The fecundity rate (i.e. number of 1^st^ instar female larvae produced per week) is in turn defined as a function of the intervention strategies:

2 $${\rm{l}}{\rm{n}}(\frac{{b}_{i,t}}{2})={\lambda }_{0}-{\lambda }_{1}IT{N}_{i,t}-{\lambda }_{2}I{M}_{i,t}-{\lambda }_{3}\varepsilon {L}_{i,t}$$

where λ_0_ determines the baseline mean weekly fecundity rate and λ_1to 3_ quantify the impacts of LLINs, IM or EL on baseline fecundity rate respectively.

Eggs hatch into larvae, a portion of which will survive to adulthood. Similar to adults, the larval survival rate was modelled as a binomial process with a weekly survival probability expressed as a function of covariates. The linear predictor of the logit transformed weekly survival rate of larvae (S_n_) was defined only as a function of density:

3 $$logit({S}_{n}(i,t))={\alpha }_{0}-{\alpha }_{1}{N}_{i,t}+{\varepsilon }_{i,t}$$

The intercept α_0_ determines the baseline mean weekly larval survival, and the density-dependence coefficient α_1_, quantifies the effect of larval density (*N*). The error term ${\varepsilon }$ accounts for extra variability unaccounted for in our experimental set-up.

The total number of female larvae (N) in each chamber is dependent on S_n_ and is calculated as half of the total number of eggs that developed through early 1^st^ and 2^nd^ larval instar stages to become readily observed 3^rd^ and 4^th^ instar. From previous semi-field studies, the interval between filial generations of *An*. *arabiensis* was approximated at 3 weeks^34^. In this model, the 2 larval stages were assumed to last one week (wk1: 1^st^ & 2^nd^ instar, wk2: 3^rd^, 4^th^ instar & pupae), with the remaining week of the generation allocated for pupal emergence (2 days) followed by host seeking, blood feeding and then egg deposition (~4 days).

The prior distributions for all baseline parameters (i.e. *β*_0_, *α*_0_, *λ*_0_), were based on published values (Table S2 in SI). To model the observation process, the likelihoods of obtaining the observed numbers of larvae and adults were generated as draws from normal distributions with mean given by their respective estimates of proportional sampling (i.e. proportion of the whole population observed by sampling). The model was fitted using the software JAGS^74^ and run for 10^6^ iterations. Further details on model fitting and evaluation are detailed in SI.

## Electronic supplementary material

Supplementary Information

## Data Availability

The dataset generated during the current study will be made freely available for access on the Dryad repository. The Full JAGS code as used for modelling analysis is provided in the Supplementary Information 10.5061/dryad.7p7s18p.
