# Supplementary material for: Mesocosm experiments reveal the impact of mosquito control measures on malaria vector life history and population dynamics
Source: Sci Rep. 2018 Sep 17;8:13949. doi: 10.1038/s41598-018-31805-8 (PMC6141522; doi:10.1038/s41598-018-31805-8)
Supplement: Supplementary file 1 — Supplementary Information [file 41598_2018_31805_MOESM1_ESM.pdf]

Supplementary information

Mesocosm experiments reveal the impact of mosquito control measures on malaria vector life-history and population dynamics

Kija Ng'habi[1,4§], Mafalda Viana[2,§], Jason Matthiopoulos[2], Issa Lyimo[1], Gerry Killeen[1,3] and Heather M. Ferguson[2*]

[1]*Ifakara Health Institute, Environmental Health and Ecological Sciences, Ifakara, United Republic of Tanzania*

[2]*Institute of Biodiversity, Animal Health and Comparative Medicine, College of Medical, Veterinary and Life Sciences, University of Glasgow, Glasgow G12 8QQ, United Kingdom*

[3]*Liverpool School of Tropical Medicine, Department of Vector Biology, Liverpool, United Kingdom*

[4]*School of Health Sciences, University of Dar es Salaam, Tanzania*

[§]equal contribution

*Corresponding Author: heather.ferguson@glasgow.ac.uk

S1. Biosphere experimental details

Mosquito breeding

From October 2012 to February 2013, blood-fed female mosquitoes identified morphologically as members of the An. gambiae complex were collected from Sagamaganga village and transported to and bred to the Ifakara Health Institute (IHI). Females were held for 24 hours with access to a 5% sugar solution before being transferred into individual cups for oviposition. Females were kept in cups for 3-5 days, after which those that oviposited were immediately taken to the IHI laboratory for species identification by polymerase chain reaction (Scott et al. 1993). Eggs from females confirmed as being An. arabiensis were pooled and released iinto aquatic habitats in each mesocosm chamber to establish populations under semi-field conditions. About 2,000 larvae were released into each mesocosm chamber the aquatic habitats over 10 to 20 introductions from independent field stock. Larval habitats were checked daily and topped up with water when necessary.

Eave louvers installation

Eave louvers were installed along the lower edge of the gap running between the roof and upper wall of the houses (Fig. 1A & B). The gap running between the roof and top of the walls (5 cms wide and 0.6 - 1.0 meters long) was covered with long-lasting insecticide treated nets (LLINs: Permanet, Deltamethrin 55gm/m2) netting material (6 x 0.6 1 m). This provides enough landing surface for the females mosquitoes prior to entering the house. Adults were expected to land on the netting surface prior to entering the hut.

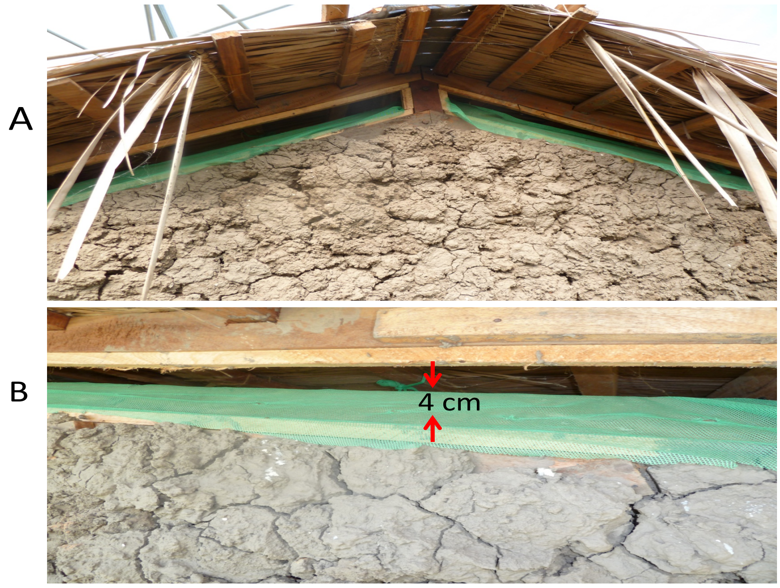

Figure S1: Photograph showing (a) eave louvers installed on the eave of the human hut in the mesocosm (b) close look of the gap and the netting material embedded on the wooden surface of the eave.

S2. State-space population model

A Bayesian population model was developed under a state-space approach to describe *Anopheles arabiensis* mosquito population dynamics, which were established inside mesocosms and exposed to different combinations of three malaria control strategies: Long lasting Insecticidal nets (LLINs), Ivermectin (IM) and eave louvres (EL). The main objective of this model was to quantify the impact of each of these control strategies on the different stages of the mosquito life cycle. A state-space model comprises a biological and an observation process. Here, the foundation of the biological process is the mosquito life cycle, i.e. adult female mosquitoes that survive to lay eggs, these eggs become larvae that undergo two larval stages before reaching adulthood and start a new cycle by laying a new generation of eggs. In turn, the observation process is based on the experimental setting and how the larval and adult data were collected.

We started by estimating the weekly survival rates of adult mosquitoes (s_p) and larvae (s_n) in each experimental compartment i at week t. These survival rates were defined through a *logit* transformation of linear predictors terms (s_p and s_n) such that:

$$s_p(i,t) = \frac{exp(S_p(i,t))}{1 + exp(S_p(i,t))} \quad (1)$$

$$s_n(i,t) = \frac{exp(S_n(i,t))}{1 + exp(S_n(i,t))} \quad (2)$$

Specifically, $S_p(i,t)$ is written as a function of the intervention strategies:

$$S_p(i,t) = \beta_0 - \beta_1 LLIN_{i,t} - \beta_2 IM_{i,t} - \beta_3 EL_{i,t} + \varepsilon_{i,t} \quad (3)$$

where, β_0 corresponds to the baseline weekly adult mosquito survival defined by an informative beta prior distribution with mean of 0.25 (see derivation of priors in Table S1) and a variance 0.01. The parameters β_1, β_2 and β_3 quantify the impact of LLIN, IM or EL, in each experimental compartment i at week t, on survival, respectively. The priors on these impacts were defined from uninformative gamma distribution with mean and variance 0.1, which allowed the impact of survival to range from no impact to elimination of the population. The uncertainty term ε was included to account for extra variability in

the survival rate within compartment i and time t associated with processes uncontrolled for by our experimental setup or other non-measured covariates and was drawn from a normal distribution with mean 0 and precision τ. In turn, τ was estimated from a uniform distribution ranging from 0 to 1.

Because our data does not include separate counts of eggs and larvae, we were not able to tease apart the impacts of the different interventions on fecundity and larvae survival rates. However, since the fecundity and larval survival stages are relatively brief and consecutive, this distinction was not deemed important from a dynamical perspective. As such, instead of describing larval survival rates as a function of the intervention strategies (as for adult survival rate), the larval survival predictor $S_n(i,t)$ was written only as function of density-dependence, where an increasing number of larvae may lead to decrease in larval survival due to predation or decreased habitat availability:

$$S_n(i,t) = \theta_0 - \theta_1 N_{i,t} + \xi_{i,t} \quad (4)$$

The coefficient θ_0 corresponds to the baseline weekly larval survival defined by an informative beta prior distribution with mean of 0.35 (see Table S1) and a variance 0.01. The density-dependence coefficient θ_1, quantifies the effect of the total number of larvae (N) present in compartment i at time t on larval survival, and was given a gamma prior with mean 0.001 and variance 0.0001. Given the size of the experimental populations, this prior allowed for density-dependence to have effects ranging from no effect or completely suppress larval survival. Similarly to adult survival, the uncertainty parameter ξ was included to account for the variability associated with the survival rates. Its prior was a normal distribution with mean 0 and precision τ. In turn, τ was estimated from a uniform distribution ranging from 0 to 1.

The total number of larvae N, however, are dependent on the survival of eggs to adulthood. Typically, eggs develop into larval stage I (n_0), and then to larval stage II (n_1) and finally pupae (n_2) before becoming full adults (p_n). As such, the total number of larvae N is a compound state variable:

$$N(i,t) = 2(n_{0i,t} + n_{1i,t} + n_{2i,t}) \quad (5)$$

This adult-egg-larval-adult development is thought to take approximately 2-3 weeks. In our model, given that our time unit is one week, we approximate this development to 3 weeks, where each larval stage lasts a full week. This development starts with adult mosquitoes p, which can be surviving adults already in the system or new adults newly emerged from larvae (p_n), reproducing at a rate $b(i,t)$ to lay 1st instar larvae that turn into pupae. The number of pupae $n_0(i, t+1)$ is then estimated from a Poisson process, such that:

$$n_0(i, t+1) \sim Poisson(b_{i,t} p_{i,t}) \quad (6)$$

and similarly to adult survival, the fecundity rate $b(i,t)$ is impacted by the intervention strategies in each compartment i such that:

$$log \frac{b(i,t)}{2} = \lambda_0 - \lambda_1 LLIN_{i,t} - \lambda 2 IM_{i,t} - \lambda_3 EL_{i,t} \quad (7)$$

where, λ_0 corresponds to the baseline weekly fecundity rate of females (hence division by 2) defined by an informative gamma prior distribution with mean of 18.75 1st instar larvae per week (equivalent to a female per capita egg production of 60 eggs, three times per week, of which only 0.25 successfully survives to 1st stage larvae; Table S1) and a variance of 25. The parameters λ_1, λ_2 and λ_3 quantify the impact of LLINs, IM and EL in compartment i at week t on the baseline fecundity rate, respectively, and were defined from uninformative gamma priors with mean and variance 0.1. This prior allowed for the impact of each intervention on fecundity rate to range from no impact to an impact large enough to cease fecundity. The pupae $n_0(i,t)$ survive to $n_1(i, t+1)$, $n_2(i, t+1)$ and young adults ready to recruit (p_n) through a consecutive binomial process with probability $s_n(i,t)$ (described in eqs 2 and 4). As the larvae become adults they join the remaining female adult population (p) and survive with the adult survival rate described in eqs. 1 and 3.

However, at each sampling event, the adults are collected destructively, which intermittently reduces the population in each compartment. Nonetheless, only a small proportion of the adult population in each compound is caught at any sampling event (defined here as 'Catchability' C_p), which only occurs every 2-4 weeks. To model this, the adult abundance data P in each compartment i at time t was generated through normal distribution:

$$P(i,t) \sim N(p(i,t)C_p, \tau_p(i,t)) \tag{8}$$

Although a binomial distribution would have been preferable here, it proved computationally challenging. Since a binomial distribution can approximate a normal distribution, we opted to model the likelihood of abundance as in eq 8. Catchability was given a beta prior distribution with mean 0.12 and variance 0.001. This prior represents the belief that only 50% of the female adults (which comprises 50% of the adult population) will be seeking a host. Of these remaining 25%, half will be seeking to bite humans and the other half to bite cattle (which were always present in the compartments). Given that human landing catches (HLC), the method used to catch adults, is only able to catch mosquitos seeking humans we estimate 12.5% of female adult mosquitos will be caught in each sampling event. We note that in weeks of no sampling there is no data informing the model and so the population status is inferred as a latent process by the life cycle in the model. In weeks of sampling, the model receives information that a certain proportion of adults was there but is culled. The precision τ_p, which is the inverse of the variance, was then defined as:

$$\tau_p(i,t) = \frac{1}{\xi C_p^2(1-C_p)p[i,t]} \tag{9}$$

where, ξC_p is the uncertainty associated with the catchability of adult mosquitoes and was defined with a gamma prior with mean 0.1 and variance 0.001. The denominator of eq. 9 corresponds to the variance of the normal distribution of eq. 8, and is weighted by the presence or absence of culling due to sampling and the number of individuals in compartment i at time t.

Similarly for larvae, the likelihood of the larval abundance data L in each compartment i at time t was generated through a normal distribution:

$$L(i,t) \sim N(\mathrm{N}(i,t)C_n, \tau_n) \tag{10}$$

This catchability C_n is defined from a beta prior distribution with mean 0.1 and variance 0.001. This prior was established from the sampling strategy: one scoop of 250ml was

taken from a homogenised 3L bucket, and all larvae in that scoop were counted. The precision τ_n, which is the inverse of the variance, was defined as:

$$\tau_n(i,t) = \frac{1}{C_n(1-C_n)N(i,t)} \tag{11}$$

The model was fit using the software JAGS (Plummer 2003) and run with 2 chains for 10^6 iterations, keeping every 100th iteration and discarding the first half, to achieve full convergence of the model. Convergence was assessed through visual inspection of the trace plots and using the Gelman-Rubin diagnostic available in the package 'coda' (Plummer et al 2012). Model fit was assessed by visual inspection of the predicted and observed densities (Fig. 5) and through a linear regression to determine the 1:1 ratio (FIg. S3).

Table S1: The priors for the baseline parameters and corresponding posteriors from the model.

**Baseline parameter**	**Literature estimate**	**Ref.**	**Prior mean [variance]**	**Posterior mean [95% CI]**
Larval survival rate	0.2-0.8 per 7-14 days	Ng'habi et al 2010	0.35 [0.01]	0.46 [0.42-0.50]
Adult survival rate	0.82 per day	Charlwood et al 1997	0.25 [0.01]	0.25 [0.18-0.35]
Fecundity rate*	60 eggs per 2.5 days	Lyimo et al 2013	18.75 [25]	25.76 [20.9-32.4]
Larval catchability	350mL from 2L	-	0.15 [0.001]	0.12 [0.10-0.14]
Adult catchability	-	-	0.1 [0.001]	0.15 [0.11-0.20]
Density dependence	-	-	0.001 [0.00001]	0.00001 [0.0003-0.00008]

*60 eggs per female. 2.5 days is the length of a gonotrophic cycle. For the prior we further assume survival from egg laying to 1st instar of 25%.

S3. JAGS code

```
model{
 for(i in 1:Ntreatments){
  #initial conditions
  n0[i,1]<-1000; n1[i,1]<-500; n2[i,1]<-500; pp[i,1]<-0; pn[i,1]<-0;

 for(t in 1:(tmax-1)){
    #larvae survival is density-dependent
    snEpsilon[i,t]~dnorm(0,tau.sn)
    logit(sn[i,t])<-sn0-sn1*nobs[i,t] + snEpsilon[i,t]
    #adult survival is treatment dependent
    spEpsilon[i,t]~dnorm(0,tau.sp)
    logit(sp[i,t])<-sp0-sp2*LLIN[i,t]-sp3*IM[i,t]-sp4*EL[i,t] + spEpsilon[i,t]

  # Survival of n0: count of larvae stage 0 (males and females)
  nD0[i,t]<-equals(n0[i,t],0)+n0[i,t]
  snD0[i,t]<-sn[i,t]-equals(n0[i,t],0)*sn[i,t]
  n1[i,t+1]~dbin(snD0[i,t], nD0[i,t])
  # Survival of n1: count of larvae stage 1
  nD1[i,t]<-equals(n1[i,t],0)+n1[i,t]
  snD1[i,t]<-sn[i,t]-equals(n1[i,t],0)*sn[i,t]
  n2[i,t+1]~dbin(snD1[i,t], nD1[i,t])
  # Survival of n2: larvae that survive this stage become adults
  nD2[i,t]<-equals(n2[i,t],0)+n2[i,t]
  snD2[i,t]<-sn[i,t]-equals(n2[i,t],0)*sn[i,t]
  pn[i,t+1]~dbin(snD2[i,t], nD2[i,t])

  # Adult survival (males and females)
  pD[i,t]<-equals(p[i,t],0)+p[i,t]
  spD[i,t]<-sp[i,t]-equals(p[i,t],0)*sp[i,t]
  pp[i,t+1]~dbin(spD[i,t], pD[i,t])
  # Total adults: surviving adults, plus new adults from n2, minus culled
  pPre[i,t]<-pp[i,t]+pn[i,t]
  cull[i,t]<-survey[t]*round(datP[i,t])
  p[i,t]<-ifelse(pPre[i,t]<=cull[i,t],1,pPre[i,t]-cull[i,t])

    # Reproduction: treatment dependent; 0.5 for females only
    b[i,t]<-0.5*exp(b0-b2*LLIN[i,t]-b3*IM[i,t]-b4*EL[i,t])
    nmu[i,t]<-b[i,t]*p[i,t]
    n0[i,t+1]~dpois(nmu[i,t])
    nobs[i,t]<-2*(n0[i,t]+n1[i,t]+n2[i,t])

  #Stochasticity in the observations
  nP[i,t]<- ifelse(pPre[i,t]<=0,1,pPre[i,t])
  pmu[i,t]<- qp*pPre[i,t]
  pprec[i,t]<-1/(Uqp*qp*(1-qp)*nP[i,t])
  datP[i,t]~dnorm(pmu[i,t],pprec[i,t])
  nL[i,t]<- ifelse(nobs[i,t]<=0,1,nobs[i,t])
  Lmu[i,t]<-qn*nobs[i,t]
  Lprec[i,t]<-1/(qn*(1-qn)*nL[i,t])
  datL[i,t]~dnorm(Lmu[i,t],Lprec[i,t])

  } #end time t loop
 } #end treatment i loop
```

```
#Priors
#"Catchability"
mu.Uqp<- 0.1
var.Uqp<- 0.001
alpha.Uqp<- pow(mu.Uqp,2)/var.Uqp
beta.Uqp<- mu.Uqp/var.Uqp
Uqp~dgamma(alpha.Uqp,beta.Uqp)

qp~dbeta(alpha.qp,beta.qp)
mu.qp<-0.1
var.qp<- 0.001
alpha.qp<- (mu.qp/var.qp)*(mu.qp-pow(mu.qp,2)-var.qp)
beta.qp<- ((1-mu.qp)/var.qp)*(mu.qp-pow(mu.qp,2)-var.qp)

qn~dbeta(alpha.qn,beta.qn)
mu.qn<- 0.15
var.qn<- 0.0001
alpha.qn<- (mu.qn/var.qn)*(mu.qn-pow(mu.qn,2)-var.qn)
beta.qn<- ((1-mu.qn)/var.qn)*(mu.qn-pow(mu.qn,2)-var.qn)

#Baseline larval survival
mu.sn0<- 0.35
var.sn0<- 0.01
alpha.sn0<- (mu.sn0/var.sn0)*(mu.sn0-pow(mu.sn0,2)-var.sn0)
beta.sn0<- ((1-mu.sn0)/var.sn0)*(mu.sn0-pow(mu.sn0,2)-var.sn0)
sn0line~dbeta(alpha.sn0,beta.sn0)
sn0<- log(sn0line/(1-sn0line))

#DD in larval survival
mu.sn1<- 0.001
var.sn1<- 0.00001
alpha.sn1<- pow(mu.sn1,2)/var.sn1
beta.sn1<- mu.sn1/var.sn1
sn1line~dgamma(alpha.sn1,beta.sn1)
sn1~dgamma(alpha.sn1,beta.sn1)

#Baseline adult survival
mu.sp0<- 0.25
var.sp0<- 0.01
alpha.sp0<- (mu.sp0/var.sp0)*(mu.sp0-pow(mu.sp0,2)-var.sp0)
beta.sp0<- ((1-mu.sp0)/var.sp0)*(mu.sp0-pow(mu.sp0,2)-var.sp0)
sp0line~dbeta(alpha.sp0,beta.sp0)
sp0<- log(sp0line/(1-sp0line))

#Treatments on adult survival
mu.int<- 0.1
var.int<- 0.1
alpha.int<- pow(mu.int,2)/var.int
beta.int<- mu.int/var.int
sp2~dgamma(alpha.int,beta.int)
sp3~dgamma(alpha.int,beta.int)
sp4~dgamma(alpha.int,beta.int)

...cont.
```

```
    #Baseline fecundity

    mu.b0<- 2.5*0.25*60*0.5
    var.b0<- 25
    alpha.b0<- pow(mu.b0,2)/var.b0n
    beta.b0<- mu.b0/var.b0
    b0line~dgamma(alpha.b0,beta.b0)
    b0<- log(b0line)

    # Treatment on fecundity
    mu.int2<- 0.1
    var.int2<- 0.1
    alpha.int2<- pow(mu.int2,2)/var.int2
    beta.int2<- mu.int2/var.int2
    b2~dgamma(alpha.int2,beta.int2)
    b3~dgamma(alpha.int2,beta.int2)
    b4~dgamma(alpha.int2,beta.int2)

    #Uncertainties
    tau.sn<- 1/pow(sigma.sn,2)
    sigma.sn~dunif(0,1)
    tau.sp<- 1/pow(sigma.sp,2)
    sigma.sp~dunif(0,1)
} #end model
```

S4. Results

Table S2: Mean larval and adult estimated densities (with 95% credible intervals) in each treatment group and at the end of each experimental phase.

	**Phase**		**Treatment**		
	**(week)**	**Control**	**LLIN only**	**IM first**	**EL first**
**Larval**	Estab. (16)	202 (178-313)	281 (253-384)	348 (317-384)	306 (278-337)
**density**	I (24)	740 (692-790)	167 (146-192)	284 (256-315)	182 (159-208)
	II (32)	1050 (993-1110)	376 (344-411)	38 (29-49)	344 (313-379)
	III (40)	1108 (1050-1172)	317 (286-350)	29 (21-40)	51 (40-65)
**Adult**	Estab. (16)	15 (12-20)	10 (7-13)	12 (9-16)	8 (6-12)
**density**	I (24)	30 (24-36)	8 (6-11)	4 (2-6)	7 (5-10)
	II (32)	24 (19-30)	10 (7-14)	1 (0-1)	9 (7-13)
	III (40)	27 (21-33)	7 (5-11)	0 (0-1)	2 (1-3)

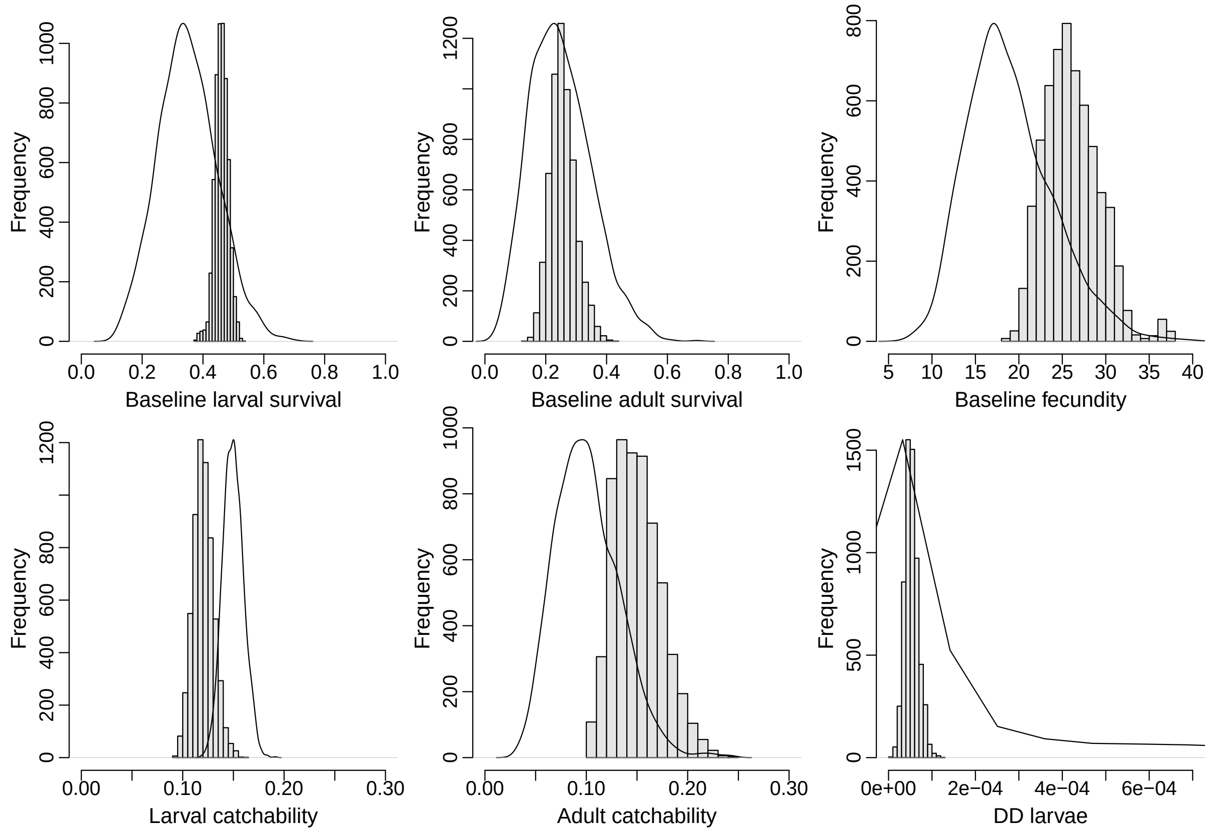

Figure S2: Prior (density) and posterior (histogram) distributions of the main parameters in the state-space model.

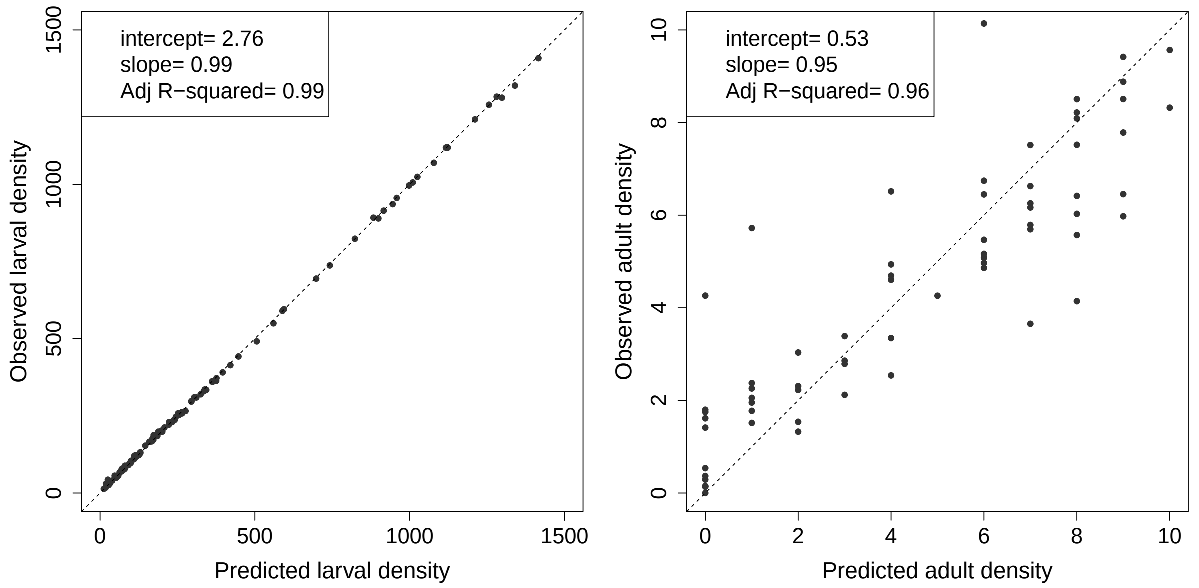

Figure S3: Goodness-of-fit. Predicted versus observed larvae (left) and adult (right) densities across all mesoscosms. Adjusted R-squared, intercept and slope values are from a linear model of the predicted against observed values. Dotted lines corresponds to 1:1 line.

References

- Plummer, M. (2003) JAGS: A program for analysis of Bayesian graphical models using Gibbs sampling. Technische Universitat Wien. - Plummer, M. et al. (2012) Output analysis and diagnostics for MCMC. CRANN Repos. Package coda'. - Nghabi, K.R. et al. (2010) Establishment of a self-propagating population of the African malaria vector Anopheles arabiensis under semi-field conditions. Malar. J. 9(1):356.

- Charlwood, J.D. et al. (1997) Survival and infection probabilities of anthropophagic anophelines from an area of high prevalence of Plasmodium falciparum in humans. Bull. Entomol. Res. 87: 455153.

- Lyimo, I.N. et al. (2013) The impact of host species and vector control measures on the fitness of African malaria vectors. Proc. Biol. Sci. 280(1754):20122823.
